# Collateral effects of antibiotic resistance in *Campylobacter jejuni*

**DOI:** 10.1093/femsle/fnag089

**Published:** 2026-07-25

**Authors:** Hadiseh Zandrajabi, Wisse van Os, Wiep Klaas Smits, Seyed Ali Mortazavi, Farideh Tabatabaei-Yazdi, Johan G C van Hasselt

**Affiliations:** Division of Systems Pharmacology and Pharmacy, Leiden Academic Center for Drug Research, Leiden University, 2333 CC Einsteinweg 55, The Netherlands; Department of Food Science and Technology, Faculty of Agriculture, Ferdowsi University of Mashhad, Azadi Square, 9177948974 Mashhad, Iran; Division of Systems Pharmacology and Pharmacy, Leiden Academic Center for Drug Research, Leiden University, 2333 CC Einsteinweg 55, The Netherlands; Experimental Bacteriology, Leiden University Medical Center, Albinusdreef 2, 2333 ZA Leiden, The Netherlands; Department of Food Science and Technology, Faculty of Agriculture, Ferdowsi University of Mashhad, Azadi Square, 9177948974 Mashhad, Iran; Department of Food Science and Technology, Faculty of Agriculture, Ferdowsi University of Mashhad, Azadi Square, 9177948974 Mashhad, Iran; Division of Systems Pharmacology and Pharmacy, Leiden Academic Center for Drug Research, Leiden University, 2333 CC Einsteinweg 55, The Netherlands

**Keywords:** *Campylobacter jejuni*, antimicrobial resistance, collateral sensitivity, collateral resistance, experimental evolution

## Abstract

*Campylobacter jejuni* is a leading cause of bacterial gastroenteritis worldwide, and resistance to major antibiotic classes is increasing. Exploiting collateral sensitivity (CS) may be an effective approach to optimize antibiotic use and mitigate the impact of resistance. In this study, we investigated CS and collateral resistance (CR) profiles in experimentally evolved *C. jejuni* mutants derived from ATCC 29428 resistant to ciprofloxacin, erythromycin, gentamicin, and tetracycline. Minimum inhibitory concentrations of thirteen antibiotics were determined in the wild-type and mutant strains to characterize collateral effects. Whole-genome sequencing was performed to identify mutations potentially underlying the observed collateral effects, and bacterial growth curves were used to evaluate the fitness of the mutants. Ciprofloxacin-resistant mutants exhibited CS most consistently to aminoglycosides, potentially explained by mutations in *gyrA, parC*, and *parE*. Gentamicin-resistant mutants showed CS to macrolides, fosfomycin, and amoxicillin. Erythromycin-resistant mutants displayed CS to aminoglycosides, albeit inconsistently across biological replicates. No CS was observed in tetracycline-resistant mutants, which showed CR to other ribosome-targeting antibiotics, potentially due to mutations in *rpsJ*. Consistent CR was observed among antibiotics of the same class. These findings demonstrate CS and CR patterns in *C. jejuni*, highlighting evolutionary trade-offs that could inform more effective treatment strategies against resistant *C. jejuni* infections.

## Teaser

Collateral effects in antibiotic sensitivity related to antibiotic resistance in *Campylobacter jejuni*.

## Introduction

*Campylobacter jejuni* is a leading cause of bacterial gastroenteritis worldwide and can cause severe gastrointestinal symptoms as well as long-term complications such as Guillain-Barré syndrome (Ge et al. 2003, Platts-Mills and Kosek 2014). Antimicrobial resistance in *C. jejuni* is increasing, driven by its inherent genetic adaptability and the widespread use of antibiotics in both clinical and agricultural settings. Specifically, the use of fluoroquinolones such as ciprofloxacin (CIP) as growth promoters and therapeutic agents in food animal production has been strongly linked to the rapid emergence and spread of resistant *Campylobacter* strains in humans (Jerome et al. 2011). As a result, macrolides, including erythromycin (ERY), are now considered first-line agents (Alfredson and Korolik 2007), but resistance to this class is also increasing, further limiting effective treatment options (Dai et al. 2020).

Leveraging collateral sensitivity (CS) may represent an effective approach to counteract resistance in *C. jejuni*. CS refers to the phenomenon where the acquisition of resistance to one antibiotic increases susceptibility to another, whereas collateral resistance (CR) represents the opposite effect (Pál et al. 2015). Designing treatment regimens that exploit these evolutionary trade-offs through antibiotic cycling or combination therapy has been proposed as a strategy to manage resistant infections (Aulin et al. 2021) and slow the further development of resistance (Imamovic and Sommer 2013).

In this study, we evaluated collateral effects in *C. jejuni* mutants with experimentally evolved resistance to CIP, ERY, gentamicin (GEN), or tetracycline (TET). We tested the susceptibility of these mutants to the other antibiotics used for experimental evolution, as well as to a broader panel including amoxicillin (AMO), azithromycin (AZI), colistin (COL), fosfomycin (FOS), imipenem, meropenem, nalidixic acid (NAL), nisin (NIS), and streptomycin (STR). These antibiotics were selected because they are commonly used in both clinical and agricultural settings and represent diverse mechanisms of action. We aimed to characterize collateral effects across these antibiotics to support the development of treatment strategies that mitigate the impact of resistance and improve treatment outcomes for *C. jejuni* infections.

## Materials and methods

### Experimental overview

The experimental workflow proceeded in four sequential stages. First, antibiotic-resistant mutants were generated through laboratory evolution by serially passaging *C. jejuni* ATCC 29428 in progressively increasing concentrations of each index antibiotic (CIP, GEN, TET, or ERY) over 10 days, with three independent biological replicates per antibiotic. Each evolution experiment produced a population-level pool of resistant bacteria rather than a single clonal isolate, better capturing the diversity of resistance mechanisms that may arise. Second, collateral effects were characterized by determining the minimum inhibitory concentrations (MICs) of thirteen antibiotics for the evolved populations and the wild-type (WT) strain, using broth microdilution according to European Committee on Antimicrobial Susceptibility Testing (EUCAST) guidelines. Third, bacterial fitness was assessed by measuring growth curves under antibiotic-free conditions and applying the Gompertz model to estimate growth rates. Fourth, whole-genome sequencing (WGS) was performed on the evolved populations to identify mutations that may underlie the observed collateral effects. The rationale for using a reference strain rather than clinical isolates was to establish a controlled, reproducible baseline for characterizing collateral effects.

### Strains, antibiotics, and media

*Campylobacter jejuni subsp. jejuni* ATCC 29428 was used as the WT ancestral strain. Bacteria were cultivated on standard Mueller–Hinton (MH) broth (VWR, The Netherlands) solidified with agar from APC Pure (United Kingdom) supplemented with 5% lysed horse blood (Sanbio, The Netherlands), and incubated in a CO₂ incubator (BINDER GmbH, Tuttlingen, Germany) under microaerophilic conditions (5% O₂, 10% CO₂, and 85% N₂) at 41°C. MIC was performed in standard MH broth (VWR) supplemented with 5% lysed horse blood and 20 mg/l β-nicotinamide adenine dinucleotide (Acros Organics) in accordance with EUCAST recommendations for Campylobacter spp. Brain–heart infusion (BHI) broth (VWR Chemicals, Leuven, Belgium) was used for the evolution experiments and growth rate measurements, as its transparency allows for optical density measurements, unlike MH broth supplemented with lysed blood. Antibiotic stock solutions were prepared according to the manufacturers’ instructions and stored at - 20°C or - 80°C. Table S1 provides detailed information on the antibiotics used in this study, including their manufacturers, catalog numbers, and solvents used for stock preparation.

### Laboratory evolution experiments

Over a period of 10 days, WT *C. jejuni* cells were exposed to progressively increasing concentrations of CIP, GEN, TET, or ERY to generate mutants resistant to these antibiotics. Three independent biological replicates (A, B, and C) were performed for each antibiotic. On day one, a bacterial suspension with an optical density at 600 nm (OD₆₀₀) of 0.5, corresponding to ~ 1.5 × 10^8^ CFU/ml, was prepared from an overnight culture. A volume of 100 µl of the suspension was inoculated into fresh BHI broth containing antibiotic concentrations of 0.5×, 1×, and 2 × MIC, to a final volume of 10 ml, resulting in a starting inoculum of ~ 1.5 × 10^6^ CFU/ml. The tubes were incubated under microaerophilic conditions at 41°C with shaking at 110 rpm. After 24 h, an aliquot from the tube with the highest antibiotic concentration that supported visible growth was diluted to an OD₆₀₀ of ~ 0.5, and 100 µl of this suspension was transferred to fresh media containing antibiotic concentrations of 1×, 2×, and 4 × the previous concentration, to a final volume of 10 ml. This process was repeated daily for 10 days (i.e. nine passages), after which the populations were passaged in antibiotic-free BHI broth for an additional 10 days to obtain stable resistant mutants, which were confirmed through MIC testing. From the final cultures, glycerol stocks were prepared and stored at − 80°C.

### Collateral effect determination

The ancestor and evolved mutants *C. jejuni* strains were plated from frozen stocks on MH blood agar and incubated for 24 h at 41°C under microaerophilic conditions. Colonies were then suspended in 10 ml BHI broth and incubated for 20–24 h under the same conditions (Rivera‐Amill et al. 2001, Malik-Kale et al. 2008). The MICs of the thirteen included antibiotics were determined for the WT and mutant strains using the broth microdilution method according to EUCAST guidelines (Giske et al. 2022). Briefly, ~ 5 × 10⁵ CFU/ml was inoculated into 96-well plates containing twofold serial dilutions of each antibiotic in MH broth containing 5% lysed horse blood and 20 mg/l beta-NAD. Plates were incubated at 41°C for 24 h under microaerophilic conditions.

Collateral effects were defined as changes in the MIC of a mutant relative to the WT strain. Decreased MIC values were classified as CS, and increased MIC values as CR, with the magnitude expressed as the log₂ fold-change relative to WT. An effect was considered biologically meaningful only when two criteria were simultaneously met: a log₂ fold-change ≥ 1 in absolute value (i.e. ≥ 2-fold MIC change) and directional consistency across biological replicates. Effects not satisfying both criteria were classified as neutral.


\begin{eqnarray*}
C{E}_M = Lo{g}_2\frac{{MI{C}_M}}{{MI{C}_W}},
\end{eqnarray*}

where CE_M_ represents the collateral effect of a mutant (CIP, TET, ERY, and GEN), and MIC_M_, and MIC_W_ are the MIC values of the mutant and WT strains, respectively.

### Growth rate measurements

A bacterial suspension of ~ 1.5 × 10⁵ CFU/ml in BHI broth was prepared as described above, and 200 µl of this suspension was transferred to each well of a 96-well plate. Optical density at 620 nm was measured hourly for 20 h at 41°C under microaerophilic conditions using a microplate reader (BioTek Instruments, Winooski, VT, USA), with 20 s of shaking before each measurement. Bacterial growth curves were analyzed using the Gompertz growth model implemented in OriginPro 2023 (OriginLab Corporation, Northampton, MA, USA). The model was fitted to the OD₆₀₀ data points between 5 h and 11 h of the growth curve to estimate the maximum growth rate, which corresponds to the slope at the inflection point of the fitted Gompertz curve. The area under the growth curve was also calculated in OriginPro 2023 using the built-in integration function. Three technical replicates were performed for each biological replicate. Relative growth rates were calculated by dividing the growth rates by the mean growth rate of the WT strain (Hasan et al. 2023, Hernando-Amado et al. 2023).

### WGS and genome assembly

DNA was isolated from overnight cultures using a DNeasy Blood & Tissue kit (Qiagen, Netherlands) according to the manufacturer’s instructions. Samples were prepared for WGS using the Illumina DNA Prep tagmentation kit and the IDT for Illumina Unique Dual Indexes. Sequencing was performed on the Illumina NextSeq2000 platform (Illumina Inc., San Diego, CA, USA) using a 300-cycle flow cell kit to produce 2×150 bp paired reads, with 1%–2% PhiX control spiked in for optimal base calling. Read demultiplexing, trimming, and running analytics were conducted using DRAGEN v3.10.12. Fastqc metrics were included for quality assessment. The QCtool pipeline (https://github.com/mtruglio/QCtool) was used to process reads, which were assembled using the SPAdes genome assembler (v3.12.0).

## Results

### Collateral effects

**Figure 1:**
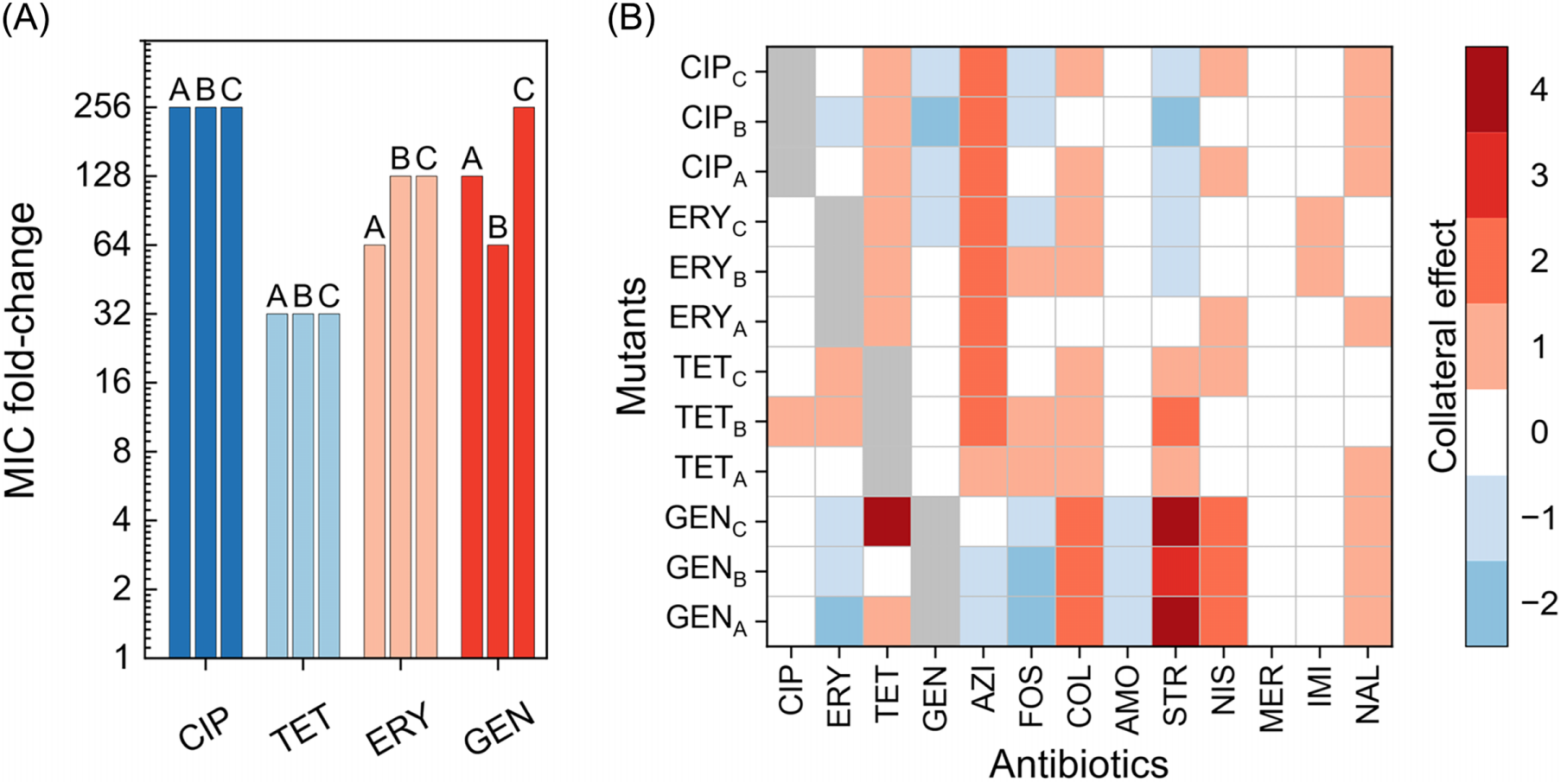
MIC fold change **(A)** and collateral effects **(B)** in the evolved mutants. Collateral effects are shown as log₂ fold-changes in MIC relative to the WT strain. The color scale indicates the direction and magnitude of collateral effects, with negative values indicating collateral resistance and positive values indicating collateral sensitivity. CIP, ciprofloxacin; ERY, erythromycin; TET, tetracycline; GEN, gentamicin; AZI, azithromycin; FOS, fosfomycin; COL, colistin; AMO, amoxicillin; STR, streptomycin; NIS, nisin; MER, meropenem; IMI, imipenem; and NAL, nalidixic acid. For antibiotic mutants, the letters A, B, and C represent biological replicates.

Antibiotic-resistant *C. jejuni* mutants were obtained through experimental evolution with CIP, GEN, ERY, or TET using three independent evolved populations per antibiotic. These resistant mutants represent population-level pools rather than single clonal isolates. All mutants showed reduced susceptibility (Fig. 1A), with MIC increases ranging from 32-fold (TET) to 256-fold (CIP). Resistance was defined as MICs exceeding EUCAST clinical resistance breakpoints for the respective antibiotics.

We evaluated collateral responses towards thirteen antibiotics to assess how resistance to one agent affected susceptibility to others (Fig. 1B). In total, twelve independently evolved resistant populations were analyzed. Overall, CIP- and GEN-resistant mutants exhibited CS most frequently across the tested antibiotics. In CIP-resistant mutants, CS was observed toward both aminoglycosides (GEN and STR) and, for two of the three biological replicates, toward FOS. In GEN-resistant mutants, CS was observed toward both of the included macrolides (ERY and AZI), FOS, and AMO. We also observed instances of CR among antibiotics within the same classes: GEN-resistant mutants also became less susceptible to STR, ERY-resistant mutants to AZI, and CIP-resistant mutants to NAL. In contrast, only CR or neutral effects were observed in TET-resistant mutants. Furthermore, CR was a common outcome across the mutant panel: nearly all mutants showed decreased susceptibility to TET. Similarly, susceptibility to COL, NIS, and NAL also decreased in nearly all mutants.

### Fitness of resistant mutants

**Figure 2:**
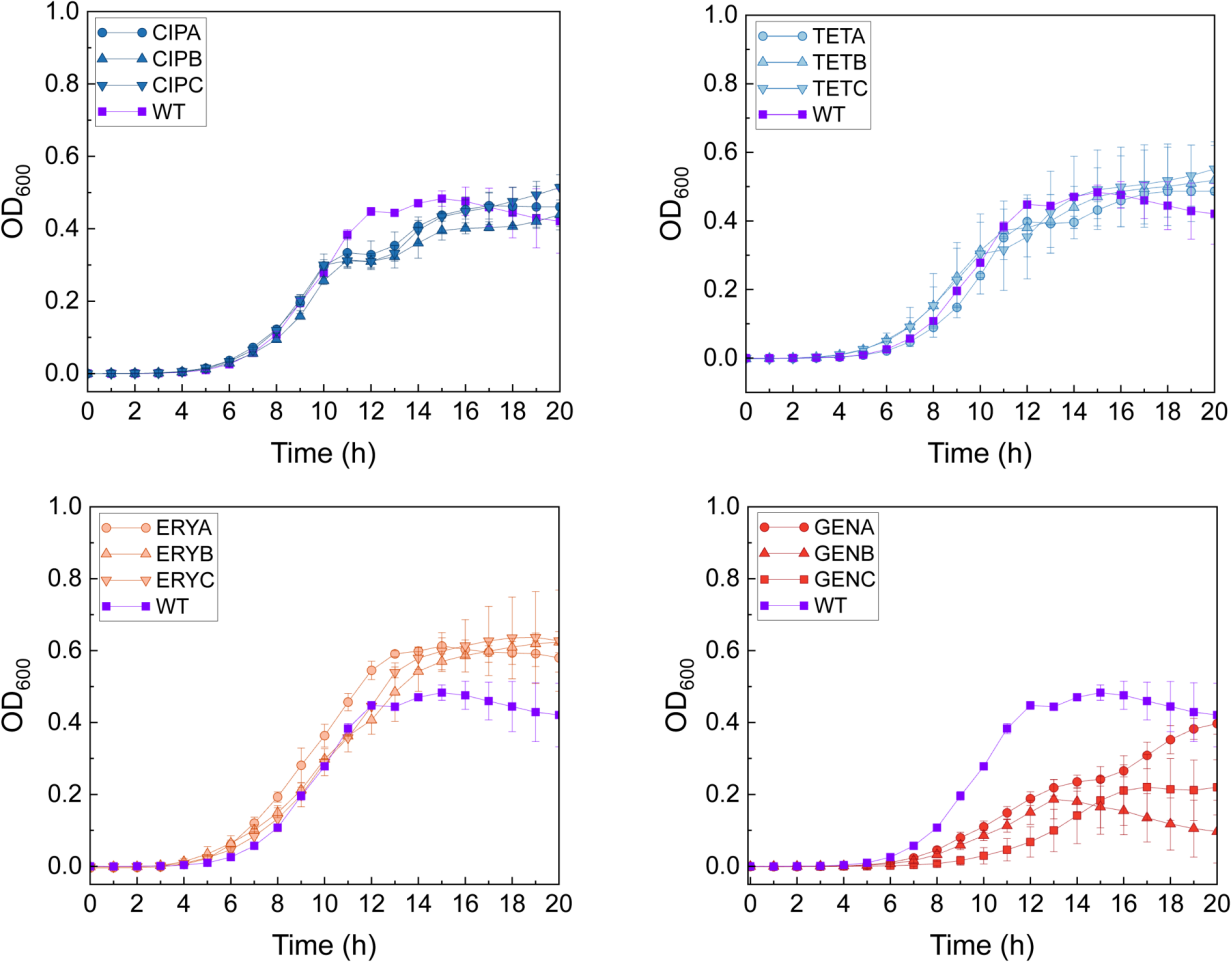
Growth rate analysis of the wild-type and antibiotic-resistant mutant strains. Growth curves for the four independently derived antibiotic-resistant mutants (CIP, ERY, TET, and GEN) over 20 h. Each curve represents the mean of three technical replicates within one biological replicate, and the figure shows three separate curves for each mutant (Biological Replicates A, B, and C).

To evaluate potential costs associated with resistance, we monitored the growth of the resistant mutants under antibiotic-free conditions (Fig. 2) and compared their growth rate (Fig. S1A) and the area under the bacterial growth curve (AUC) with the WT strain (Fig. S1B). Reduced growth rate was used as an indicator of fitness costs. A potential cost in growth rate was observed for GEN-resistant mutants, which exhibited lower growth rates and AUC values than the WT strain. CIP-resistant mutants showed a transient reduction in growth toward the end of the exponential growth phase.

### Whole-genome sequencing

**Figure 3:**
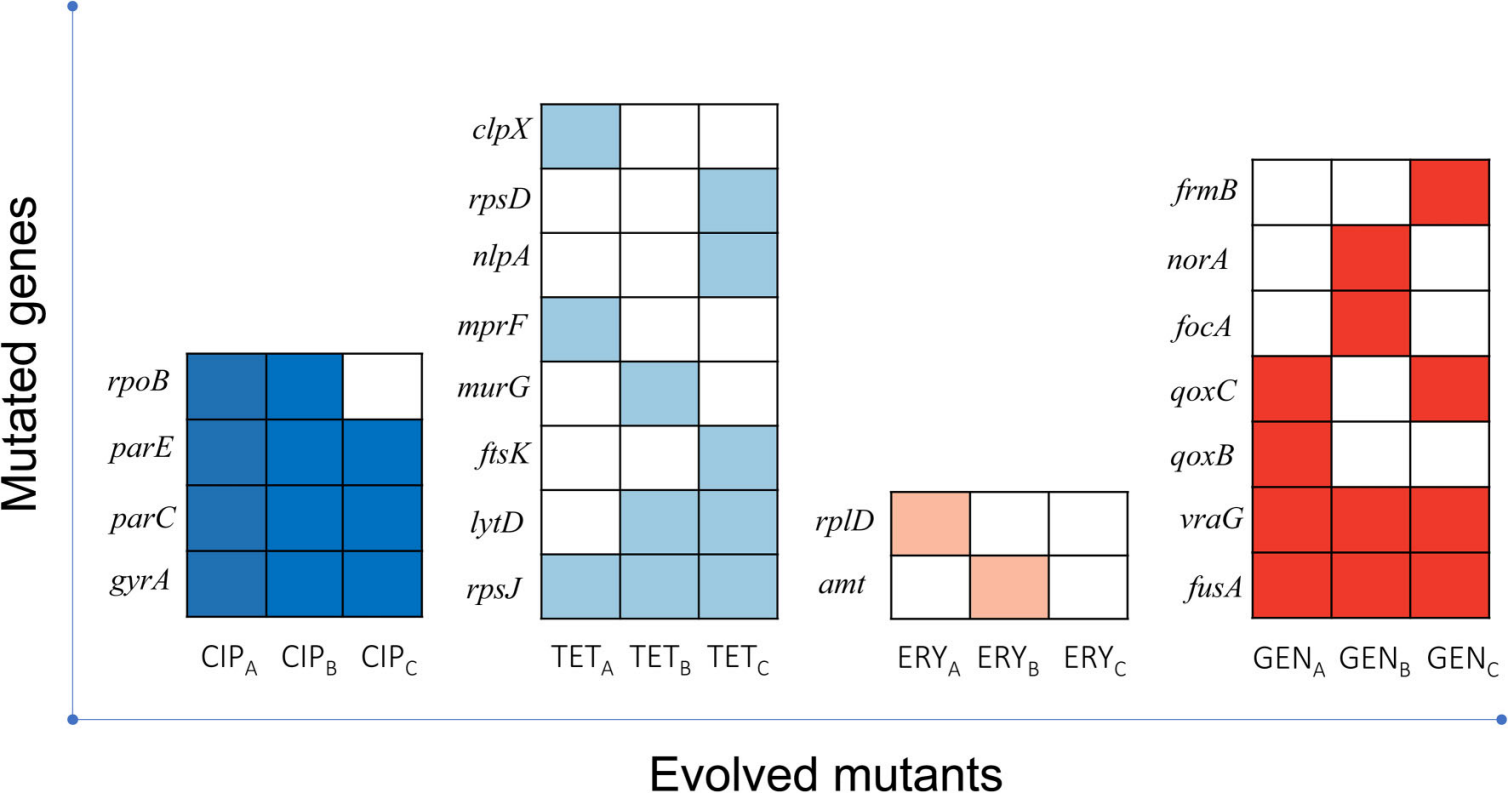
Selected mutations identified in the evolved mutants. Each antibiotic label (A, B, and C) represents three independent biological replicates generated through experimental evolution under the indicated antibiotic. CIP, ciprofloxacin; ERY, erythromycin; TET, tetracycline; GEN, gentamicin; AZI, azithromycin; FOS, fosfomycin; COL, colistin; AMO, amoxicillin; STR, streptomycin; NIS, nisin; MER, meropenem; IMI, imipenem; and NAL, nalidixic acid.

To identify mutations that are potentially linked to collateral effects, we performed WGS. Only mutations with known or putative functional relevance, such as those encoding target proteins or enzymes, were considered for further analysis. Mutations in intergenic regions or genes annotated as hypothetical proteins were excluded. Mutations identified in genes are shown in Fig. 3. Across all CIP mutants, we identified 10 mutations, including in *gyrA* and *parC*, which encode subunits of DNA gyrase and topoisomerase IV known to confer fluoroquinolone resistance. TET-resistant mutants carried 15 mutations, including key alterations in the *rpsJ* gene, which encodes a ribosomal protein implicated in TET binding. ERY-resistant mutants exhibited 24 mutations, notably in *amt* and *rplD*, the latter encoding a ribosomal protein linked to macrolide resistance. GEN mutants had 21 mutations, including changes in *vraG* and *fusA*, genes involved in membrane transport and translation processes associated with aminoglycoside resistance.

## Discussion

This study characterized collateral effects arising from experimentally evolved resistance to four key antibiotic classes in *C. jejuni*. These findings provide insights into which antimicrobials CS might or might not represent a viable route to inform treatment strategies, helping to mitigate the growing burden of antimicrobial resistance in this pathogen.

CIP-resistant mutants showed CS to aminoglycosides (STR and GEN) (Hasan et al. 2023, Hernando-Amado et al. 2023) and FOS and CR to TET, AZI, and NAL. This CS profile may be associated with mutations in the quinolone resistance-determining regions of *gyrA* and *parC*. Such mutations, beyond preventing quinolone binding, can alter DNA supercoiling and global gene expression (Smith and Fratamico 2010, Han et al. 2012, Liakopoulos et al. 2022), potentially increasing aminoglycoside uptake, as reported in other species (Chen et al. 2007, Niu et al. 2022). Given that fluoroquinolones are no longer recommended as first-line therapy for *C. jejuni* infections due to widespread resistance (Alfredson and Korolik 2007), our findings suggest that aminoglycosides could serve as effective treatment options against fluoroquinolone-resistant strains. Conversely, the observed CR to AZI and TET indicates these antibiotics may be less suitable in this context.

Mutants selected with ERY, the current first-line treatment for Campylobacteriosis (Alfredson and Korolik 2007), did not exhibit consistent CS, although some replicates showed increased susceptibility to the aminoglycosides and FOS. These mutants harbored a mutation in *rplD* (encoding the L4 ribosomal protein), a known determinant of macrolide resistance (Lynch et al. 2020). Notably, no reduction in growth rate was observed for these mutants, despite this mutation being often associated with fitness costs (Pereyre et al. 2004, Zaman et al. 2007). This may be explained by compensatory evolution, such as the observed mutation in the ammonium transporter (*amt A216T*), which may have helped offset fitness costs. The absence of observed CS in ERY-resistant mutants is concerning, as it suggests limited potential for antibiotic cycling strategies that exploit CS to combat infections resistant to the current first-line treatment. However, the absence of cross-resistance to aminoglycosides is relevant, particularly as macrolide resistance increases (Dai et al. 2020). Our data suggest that aminoglycosides may remain effective against macrolide-resistant strains, as they are not substrates for macrolide efflux pumps like CmeABC (Sharifi et al. 2021).

GEN-resistant mutants demonstrated broad CS, with increased susceptibility to antibiotics from multiple classes, including macrolides (ERY and AZI), FOS, and AMO. In contrast, they exhibited CR to other cationic drugs, including STR and COL, which may be linked to mutations identified in the *vraG* ABC transporter and *qox* respiratory genes (Laborda et al. 2022, Spreacker et al. 2022). Although GEN is not a primary treatment for *C. jejuni*, its widespread use in agriculture and the rise of aminoglycoside resistance in other pathogens underscore the need for a better understanding of the consequences of aminoglycoside resistance. GEN-resistant mutants incurred a substantial fitness cost, consistent with mutations in *fusA* (encoding elongation factor G), which are known to slow translation and reduce fitness (Bielecki et al. 2012, Wieland et al. 2022). This suggests that GEN-resistant strains may be less virulent or competitive.

TET-resistant mutants exhibited no CS to any antibiotic tested but instead developed broad CR, including to macrolides and STR, with no measurable fitness cost. This multidrug-resistant profile appears linked to a combination of mutations identified in our WGS analysis, including ribosomal changes (e.g. in *rpsJ*) and cell envelope modifications (e.g. in *mprF*), which have been reported to confer resistance across multiple drug classes (Hu et al. 2005, Villa et al. 2014, Ernst et al. 2018). Given the widespread use of TETs in agriculture, a major reservoir for *C. jejuni*, these findings are concerning. TET selection can promote the emergence of highly fit, multidrug-resistant strains that lack evolutionary trade-offs that can be exploited by cycling strategies, posing challenges to both clinical treatment and antibiotic stewardship.

Although our work is presently limited to a single reference strain of *C. jejuni*, which constrains the generalizability of our findings, we make several important observations. CS and CR patterns can vary between genetic backgrounds, as it has been shown that strain-dependent variation can influence CS outcomes (Podnecky et al. 2018, Nichol et al. 2019). In this context, the number of independently evolved mutants for each antibiotic was limited. While our WGS data provide insights into candidate genetic changes associated with the observed collateral effects, experiments with a larger number of independent evolutionary replicates will be required to robustly establish their causal contribution. These considerations are particularly relevant when evaluating the broader applicability of our findings. Future research should validate the observed collateral response patterns across a diverse panel of clinical *C. jejuni* isolates. *In vivo* studies are also needed to confirm whether the observed CS, particularly the CIP-aminoglycoside relationship, translates into therapeutic efficacy. Finally, mechanistic studies are needed to fully elucidate the pathways underlying these collateral effects.

In conclusion, our study reveals distinct patterns of CS and CR in *C. jejuni* mutants with experimentally evolved resistance to clinically relevant antibiotics. Specifically, the observed CS in CIP-, GEN-, and ERY-resistant mutants suggests that strategic antibiotic pairing or cycling, such as using aminoglycosides for CIP-resistant strains and macrolides or β-lactams for GEN-resistant strains, may enhance therapeutic efficacy. These insights may serve as a framework for developing evidence-based strategies to manage resistance and optimize antibiotic use in both clinical and veterinary contexts, particularly by designing antibiotic therapies that exploit the identified evolutionary trade-offs (Aulin et al. 2021) to improve treatment outcomes in resistant *C. jejuni* infections.

## Supplementary Material

fnag089_Supplemental_File
