# Supplementary material for: Collateral effects of antibiotic resistance in Campylobacter jejuni
Source: FEMS Microbiol Lett. 2026 Jul 25;373:fnag089. doi: 10.1093/femsle/fnag089 (PMC13426002; doi:10.1093/femsle/fnag089)
Supplement: fnag089_Supplemental_File [file fnag089_supplemental_file.docx]

**Exploring Collateral Effects of Antibiotic Resistance in *Campylobacter jejuni***

Hadiseh Zandrajabi^1,2^, Wiep Klaas Smits^3^, Seyed Ali Mortazavi^2^, Farideh Tabatabaei-Yazdi^2,*^, J. G. C. van Hasselt^1,*^

^1^ Systems Pharmacology and Pharmacy, Leiden Academic Center for Drug Research, Leiden University, Einsteinweg 55, Leiden, 2333 CC, The Netherlands

^2^ Department of Food Science and Technology, Faculty of Agriculture, Ferdowsi University of Mashhad, Mashhad, Iran

^3^ Leiden University Medical Center, Leiden, The Netherlands

Table S 1. Antibiotics used in this study, with their solvent and solubility (mg/mL) based on manufacturer data.

| Drug type | Brand | CAS# | Solvent | Solubility | Storage °C |
| --- | --- | --- | --- | --- | --- |
| Gentamycin | Chem-Impex | 1405-41-0 | Water | 50 mg/mL | -20 |
| Streptomycin | Chem-Impex | 3810-74-0 | Water | 50 mg/mL | -20 |
| Amoxicillin trihydrate | Alfa Aesar | 61336-70-7 | 1 M NH4OH | 10 mg/mL | -20 |
| Nalidixic acid | Alfa Aesar | 389-08-02 | Water | 0.1 mg/mL | -20 |
| fluoroquinolone | Chem-Impex | 85721-33-1 | 0.1 M HCl | 25 mg/mL | -20 |
| Tetracycline hydrochloride | Chem-Impex | 64-75-5 | Water | 50 mg/mL | -20 |
| Azitromycin | Sigma-Aldrich | 117772-70-0 | Ethanol | 1 mg/mL | -20 |
| Erythromycin | Chem-Impex | 114-07-8 | Ethanol | 2 mg/mL | -20 |
| Colistin | Cayman Chemical | 1404-26-8 | Water | 50 mg/mL | -20 |
| Fosfomycin disodium | Biosynth | 26016-99-9 | Water | 40 mg/mL | -20 |
| Nisin | Biosynth | 1414-45-5 | Water | 57 mg/mL | -20 |
| Imipenem monohydrate | Biosynth | 74431-23-5 | Water | 10 mg/mL | -80 |
| Meropenem Trihydrate | Biosynth | 119478-56-7 | Water | 8 mg/mL | -80 |


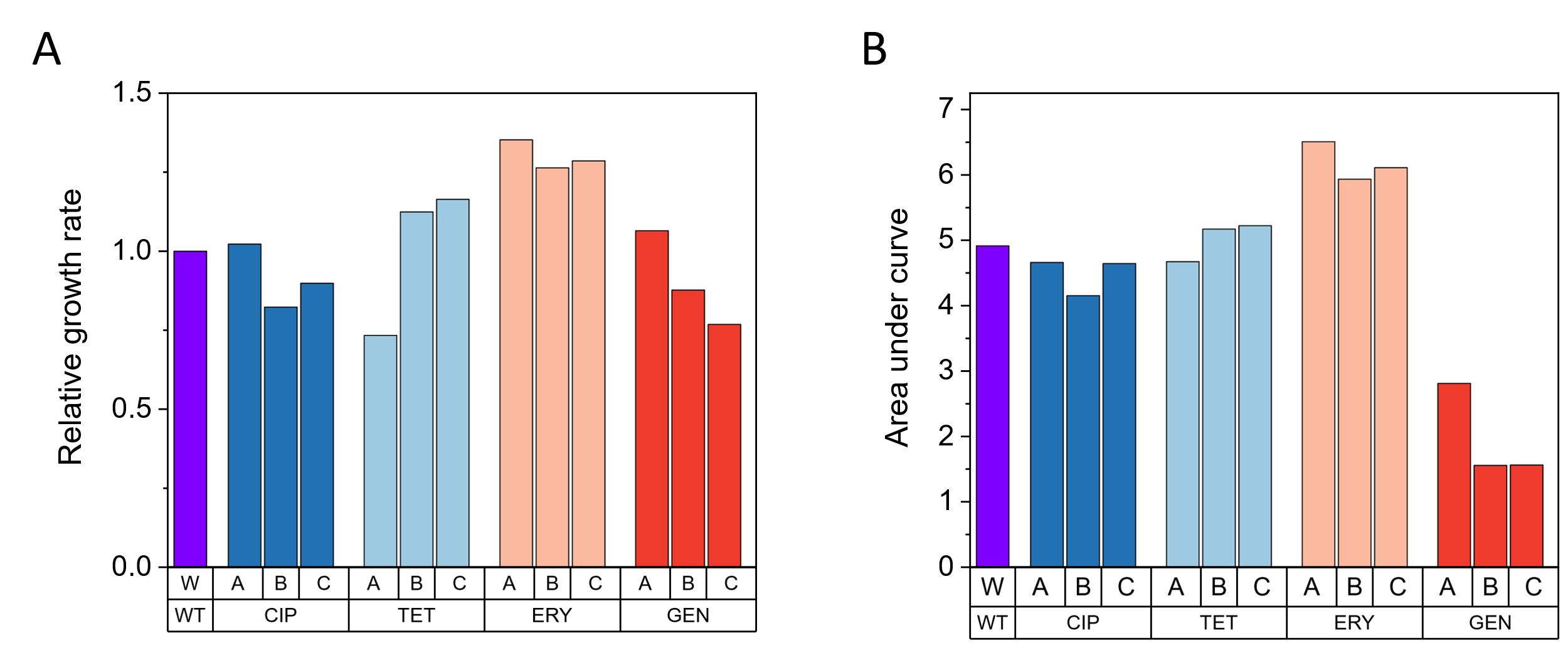


Figure S 1. (**A**) Growth rate of the mutants relative to the mean growth rate of the wild-type strain. (**B**) The area under the bacterial growth curves. CIP, ciprofloxacin; ERY, erythromycin; TET, tetracycline; GEN, gentamicin; AZI, azithromycin; FOS, fosfomycin; COL, colistin; AMO, amoxicillin; STR, streptomycin; NIS, nisin; MER, meropenem; IMI, imipenem; NAL, nalidixic acid. One-sample t-tests against the WT mean (n=3 per group) revealed a significant increase in both growth rate (p=0.008) and AUC (p=0.019) for ERY-evolved mutants, and a significant reduction in AUC for GEN-evolved mutants (p=0.021), while CIP- and TET-evolved mutants showed no significant difference from WT.
